# In-situ Monitoring on Micro-hardness of Laser Molten Zone on AISI4140 Steel by Spectral Analysis

**DOI:** 10.1038/s41598-019-55559-z

**Published:** 2020-03-06

**Authors:** Siyu Wang, Yichen Wang, Changsheng Liu, Jyoti Mazumder

**Affiliations:** 1 Key Laboratory for Anisotropy and Texture of Materials (Ministry of Education) Northeastern University, Shenyang, 110819 China; 2 Material Science and Engineering Department, University of Michigan, Ann Arbor, Michigan 48109 USA

**Keywords:** Mechanical properties, Characterization and analytical techniques

## Abstract

The real-time monitoring technology plays a significant role in the field of laser aided manufacturing. It not only ensures the product quality, but also saves time and expenditure on the subsequent testing. To develop a method to monitor the properties of laser molten zone, in this paper, the AISI4140 steel samples were melted by laser with different parameters. At the same time, the plasma spectra were detected during real-time laser processing. The evolutions for both emission spectra and hardness of molten zone were researched in this work. To correlate the intensity of spectral line with the hardness of molten zone, the method of dimensionless analysis was used in this experiment. As the results shown, in a dimensionless system, there was a linear correlation between dimensionless micro-hardness of molten zone (H*) and dimensionless laser energy density(ln(δ*)); the dimensionless micro-hardness could be expressed by a piecewise function using dimensionless intensity of Fe I spectral lines(I*), dimensionless velocity(v*) and dimensionless laser energy density as variables; depending on the quantitative relation among all dimensionless, a monitoring system of hardness of molten zone was established; by testing under different parameters of laser processing, the mean error of prediction is lower than 3.1%. It means the emission spectroscopy can be a potential way to monitor the properties of parts prepared by laser processing.

## Introduction

The laser, with high energy density, is widely used for materials processing such as laser cutting, laser welding, laser cladding and rapid prototyping process after it was invented in 1960s^1^. Due to the characteristic of laser manufacture and the quality requirements of related product, technologies about real-time monitoring have been researched and applied for the field of laser manufacture^2–4^. Among all kinds of monitoring methods, the emission spectra^5^, generated during interaction between laser and materials, is a significant signal to reflect the properties of laser molten pool^6–8^. For the laser induced spectra, it was usually applied to calculate the plasma temperature and electron density in the early researches^9–13^, which was contributed to the energy absorption and energy transition during laser processing^14,15^. As the research continues, the technology of laser induced spectroscopic diagnostics has been used in laser manufacture process, Song and Mazumder^16^ used emission spectra to calculated element concentration in Fe-Cr cladding layer, the accuracy of the measured Cr concentration was within 2.78 at.% and the average resolution was around 5.21 at.%, moreover, they found the phase transition in the cladding layer could make odd variations in the spectra^17^. Liu^18,19^ proved that there was the correlation between the electron temperature and the stability during both the welding and cladding process. Bi^20–22^ focused on the correlation between the spectrum signal of the molten pool and the quality of cladding layers. Besides these, the variation of data collected in the laser induced spectroscopy, such as peak intensity, peak width, plasma temperature and electron density can be set as spectral signals to monitor the laser welding quality and detect common defects, such as crack, oxidation and lack of penetration^23–26^.

According to the published reports, it can be found most researches on real-time spectroscopic diagnostics are focused on predicting the macro quality of products. The relevant studies on the monitoring of mechanical properties are rare. It means that the performance of products fabricated by laser processing should still be tested by the traditional mechanical measurements. In this paper, to establish a monitoring system to predict mechanical properties of laser molten zone, AISI4140 steel was melted by disk laser with different parameters, while the emission spectra were collected in a real laser processing. The micro-hardness variation of molten zone was studied in this work and the intensity variation of peaks in spectra was analyzed for all the samples. To establish the correlation among the variables with different process parameters, a dimensionless system was established in this research. The dimensionless laser energy density was deduced in the dimensionless analysis, in addition, the hardness and spectral intensity were processed by normalized treatment. Finally, the correlations among dimensionless spectral intensity, dimensionless micro-hardness and dimensionless laser energy density were established by the non-regression method.

## Experiment and Methods

### Sample preparation

The AISI4140 steel plates were used in this experiment. The original size of the plate was 6 inch × 2 inch × 0.25 inch (15.24 cm × 5.08 cm × 0.64 cm), and the chemical component of AISI4140 steel is shown in Table 1. The original substrates were annealed with a box furnace (Thermo Scientific Lindberg/Blue M) before experiment, they were heated to 860 °C with the heating rate of 20 °C/min, then dwell at 860 °C for 1 hour, finally, they were cooled inside the furnace to room temperature.

**Table 1:** Chemical component of AISI 4140 steel.

Element	C	Mn	Si	Mo	Cr	P	S	Fe
Content	0.38–0.43	0.75–1.00	0.15–0.30	0.15–0.25	0.80–1.10	0.035max	0.040	Bel.

The original plates were cut into small size as 1 inch × 2 inch × 0.25 inch (5.08 cm × 2.54 cm × 0.64 cm) after heat treatment, and then the surface of samples was milled with #100 abrasive paper and cleaned with non-water ethanol to eliminate the dirty spots and the oxidation layer generated during heat treatment. The surface of pre-processing samples was melted by a disk laser (TRUMPF laser HDL 4002, German). This he laser model has continuous wavelength (1030 nm). The beam diameter was 0.8 mm, which was 6 mm lower than focus point plane. The laser scanning direction was followed along with the long dimension of samples. 5 kinds of laser power (1000 W, 1200 W, 1400 W, 1600 W and 1800 W) and 4 kinds of laser scanning speed (2.5 mm/s. 5 mm/s, 10 mm/s and 20 mm/s) were used in this experiment, which meant there were 20 groups of laser parameters in total. The argon gas flowing perpendicular to the surface was used to shield the samples. The flow rate of shielding gas is 15 SCFH (7.06 m^3^/min), which played a role of protection of molten pool. The laser processing was repeated for 3 times under the same laser parameters.

### Optical system

**Figure 1:**
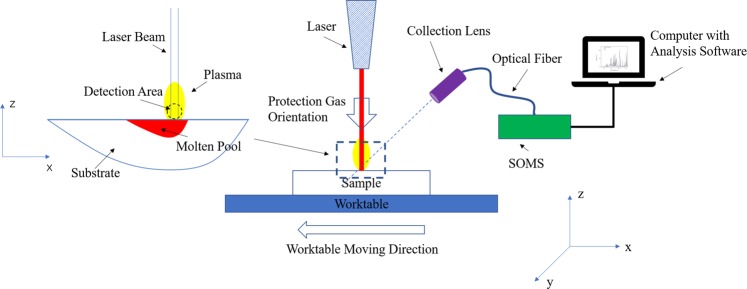
Schematic diagram of the experimental system.

The experimental schematic diagram is shown in Fig. 1. The SOMS (Smart Optical Monitoring System) was designed and produced by Sensigma LLC. (Ann Arbor, Michigan, USA). The detected orientation was parallel to the surface of samples. The detected point for the plasma signal, with a parallel incident angle, was 2 millimeters above the substrate and 70 millimeters away from the laser beam. This viewing method avoided the interference from the blackbody radiation from the molten pool^16,17^. The spectrometer had an entrance aperture of 10-micron width, a holographic UV grating with a groove density of 4800 per millimeter, and a 2048-element CCD-array detector. The resolution of the spectrometer was 0.05 nm. The range of wavelength was from 340 nm to 430 nm and the integration time of spectra was 5 ms in this experiment.

For each sample, the number of emission spectra increased with the decline of laser scanning speed. The peaks intensity had large fluctuation when the laser passed the edge of substrate. Considering the stability of spectra and amount of data group, only 50 spectra in the middle part of data group were selected for analyzing in next step, which meant 150 spectra were picked up for each sample in total.

**Figure 2:**
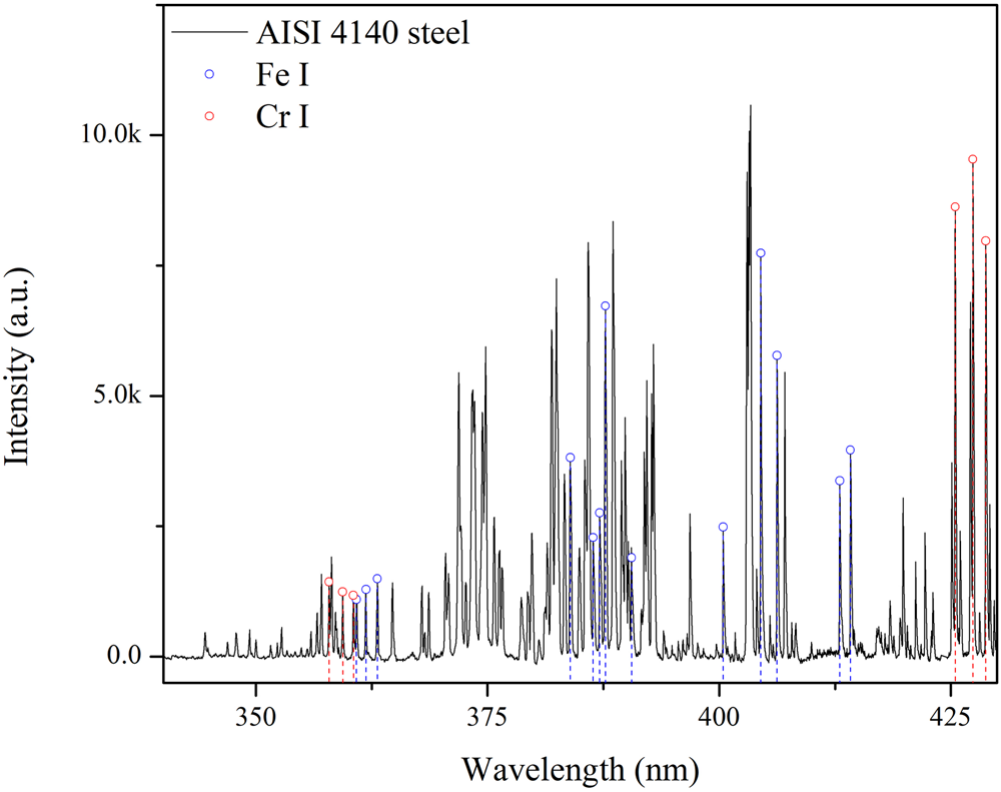
The representative spectrum of the AISI4140 sample.

In this research, the spectral lines excited by the main alloy elemental (Cr) and the matrix elemental (Fe) were selected and measured for each sample. The typical spectrum of AISI4140 steel in the range of 340 nm–430 nm was shown in Fig. 2. By comparison with the wavelength of spectral lines offered in NIST (National Institute for Standards and Technology) databases^27^, 13 Fe I spectral lines and 6 Cr I lines were picked out from the spectrum. The parameters of each spectral lines are shown in Table 2.

**Table 2:** The parameters of analysis lines.

	Wavelength λ (nm)	degeneracy and transition probability g_ij_A_ij_ (s^−1^)	lower energy level E_i_ (cm^−1^)	upper energy level E_j_ (cm^−1^)
Fe I	361.0159	5.90E + 07	22650.416	50342.129
Fe I	361.8768	5.05E + 08	7985.785	35611.625
Fe I	363.1462	4.65E + 08	7728.060	35257.324
Fe I	383.4222	4.08E + 08	12968.554	38995.736
Fe I	384.1047	4.65E + 07	8154.714	34017.103
Fe I	386.5522	5.25E + 07	7985.785	33801.572
Fe I	387.8572	1.85E + 07	704.007	26479.381
Fe I	390.6479	2.50E + 06	888.132	26479.381
Fe I	400.5241	1.02E + 08	12560.934	37521.161
Fe I	404.5811	7.76E + 08	11976.239	36686.176
Fe I	406.3593	4.66E + 08	12560.934	37162.746
Fe I	413.2057	8.26E + 07	12968.554	37162.746
Fe I	414.3867	1.20E + 08	12560.934	36686.176
Cr I	357.8704	8.10E + 08	0.000	27935.241
Cr I	359.3502	1.33E + 09	0.000	27820.197
Cr I	360.5345	1.05E + 09	0.000	27728.811
Cr I	425.4352	1.58E + 08	0.000	23498.815
Cr I	427.4812	2.84E + 08	0.000	23386.341
Cr I	428.9731	2.15E + 08	0.000	23305.002

For one spectrum, to attenuate the negative effect from a single spectral line, the profile of spectral line was fitted by the Voigt function firstly^28^, and then the mean intensity of spectral lines excited by the same elemental atom was calculated by:

1 $${I}_{mean}=\frac{{I}_{{\lambda }_{1}}+{I}_{{\lambda }_{2}}+\ldots +{I}_{{\lambda }_{i}}+\ldots +{I}_{{\lambda }_{n}}}{n}$$

where ${{\rm{I}}}_{{{\rm{\lambda }}}_{{\rm{i}}}}$ is the intensity of a spectral line with thewavelength of λ_i_.

Then the average-value of *I*_*mean*_ for the data sets was calculated by.

2 $$\mathop{{I}_{mean}}\limits^{-}=\frac{{I}_{mean}^{1}+\ldots +{I}_{mean}^{j}+\ldots +{I}_{mean}^{m}}{m}$$

Where ${{\rm{I}}}_{{\rm{mean}}}^{{\rm{j}}}$ is the mean spectral intensity calculated by j^th^ spectrum. In this experiment, m is 150.

The relative mean intensity (*I*_*relative*_) was the ratio of experimental data to the minimum spectral $\bar{{I}_{mean}}$. In this experiment, the minimum spectral $\bar{{I}_{mean}}$ was detected under 1000 W of laser power and 20 mm/s of laser scanning speed.

### Microstructure and hardness

After laser processing, the specimens of molten zone were cut from the middle part of sample plates, they were perpendicular to the laser scanning orientation. The cross-section of specimens was grilled with the abrasive papers from #120 to #2000, then they were polished with 3 μm polish paste, cleaned with anhydrous ethanol and dried with hot air, finally, they were etched by electrolyte solution made up with 96 vol% alcohol and 4 vol% nitric acid for 5 s. The microstructures of the molten pool were observed by scanning electron microscope (Tescan MIRA3 FEG SEM). The hardness of samples was measured with a Vickers micro-hardness tester (Clark CM-400AT). The load was 500 g and the dwell time was 15 seconds. For each sample, the hardness testing orientation was from the center to the edge of molten pool; there were three testing rows for one sample and there were 5 testing points in each row; the distance between each row was 100 μm and the spacing between each point was around 1/8 depth of molten zone. Finally, there were 15 test point in a molten zone for each sample in total. The final micro-hardness used in this experiment was the mean calculated with 15 points for each sample.

## Results

### Characteristic of molten zone

**Figure 3:**
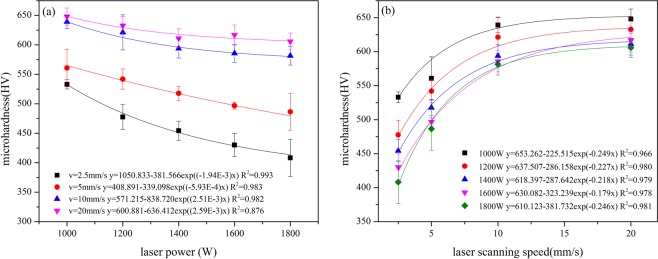
The correlation between micro-hardness of molten zone and the laser parameters (**a**) micro-hardness and laser power (**b**) micro-hardness and laser scanning speed.

The correlations between laser parameters and micro-hardness of molten zone are shown in Fig. 3. The hardness of molten zone reduces with the increase of laser scanning velocity, rather, it enhances with the reduction of laser power. Moreover, when the laser power is higher than 1400 W or the laser scanning speed is lower than 5 mm/s, the variation range for the hardness becomes significant. In addition, the relationships among hardness of molten zone, laser power and laser scanning speed can be expressed by exponent dependence function.

**Figure 4:**
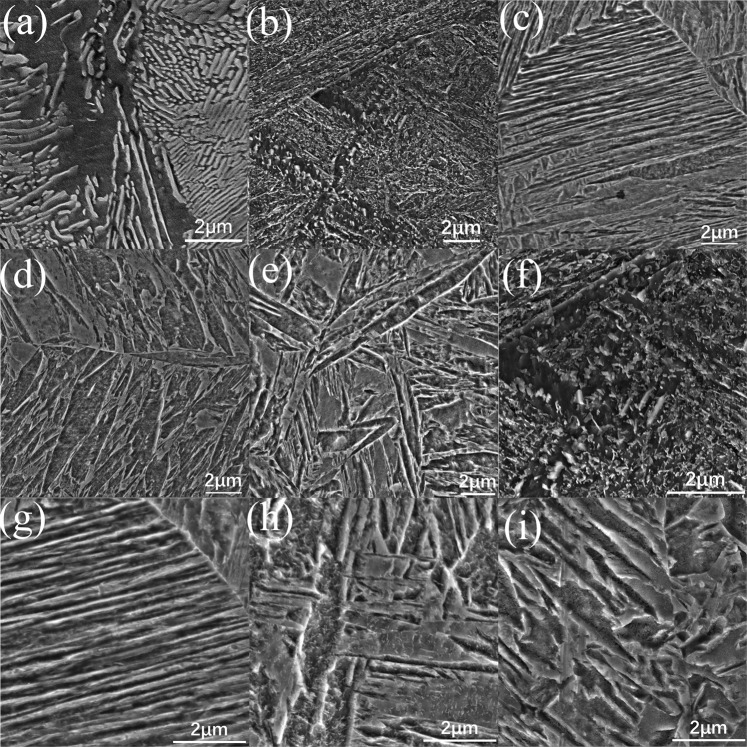
Representative microstructure of molten zone with different laser parameters (**a**) original microstructure of substrate; (**b**), (**f**) 1800 W 2.5 mm/s; (**c**), (**g**) 1600 W 5 mm/s; (**d**), (**h**) 1400 W 10 mm/s; (**e**), (**i**)1000 W 20 mm/s.

The typical microstructures of molten zone are shown in Fig. 4. It can be observed the microstructure is varied with the change of laser parameters. Figure 4(a) is the microstructure of original substrate. It is composed of Ferrite and Pearlite. When the laser power is 1800 W and the laser scanning speed is 2.5 mm/s, the microstructure of molten zone is demonstrated in Fig. 4(b,f), it is a kind of blocky microstructure with small particles on its surface. With the increase of laser power or the reduction of laser scanning speed, both of plumose microstructure and leaf-like microstructure can be found in the molten zone, which are shown in Fig. 4(c,d). In the plumose microstructure, it can be found that these sheaves contain several lath-like structures that are approximately parallel to each other, which is shown in Fig. 4(g); for the leaf-like microstructure, some micro particles are separated out from the leaf-like substrate, which is shown in Fig. 4(h). With the further change of laser parameters, the plumose microstructure can be found rarely in the molten zone, however, some aciculiform phases was observed in the edge of molten zone, which is shown as Fig. 4(e). Compared with the morphology of phase shown in Fig. 4(h), the surface of aciculiform phases shown in Fig. 4(i), is smoother and no precipitate can be found on these phases. Depending on the typical morphology of microstructure in steel and the continuous cooling transformation diagram^29–31^, it can be point that. The microstructure shown in Fig. 4 is Ferrite and granular Bainite (b,f), upper Bainite (c,g), lower Bainite(d,h) and Martensite (e,i) respectively.

### Analysis of spectra data

**Figure 5:**
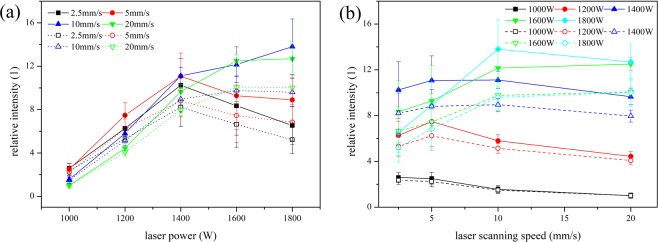
The correlation between relative mean intensity of spectral lines and laser parameters (solid points: Cr I, empty points: Fe I) (**a**) relative mean intensity and laser power; (**b**) relative mean intensity and laser scanning speed.

The correlations between laser parameters and mean intensity of spectral lines are shown in Fig. 5. When the laser scanning speed is higher than 10 mm/s, the mean intensity of Cr I spectral lines increase with the laser power changing from 1000 W to 1800 W, correspondingly, for that of Fe I spectral lines, when the laser power is higher than 1400 W, the increase rate becomes slight. When the laser scanning speed is lower than 5 mm/s, for both Cr I spectral lines and Fe I spectral lines, the mean intensity keeps growing up until the laser power reach to 1400 W, and then the mean intensity declines with the continuous increase of laser power. It can be seen from Fig. 5(b), for both Cr I and Fe I spectral lines, the varied trends between relative mean intensity and laser scanning speed are similar. When the laser power is 1000 W, the relative intensity reduces with the increase of laser scanning speed, however, when the laser power is higher than 1200 W, the relative intensity decreases after the original increase. When the laser power is 1200 W, the highest relative intensity appears at 5 mm/s, while the position of highest intensity shifts to be at10 mm/s while the laser power rises higher than 1400 W.

## Discussion

The main reason is the phase transition is the function of change of cooling rate under different laser parameters, and the cooling rate can be shown as^1^:

3 $$\frac{\partial T}{\partial t}=2\pi k\frac{v}{Q}{(T-{T}_{0})}^{2}$$

where *v* is transition speed, *Q* is absorbed power, *k* is thermal conductivity and *T*_0_ is initial temperature.

In this experiment, the elements concentration is same for each sample, so the starting temperature for austenite phase transformation is same for all the samples. It means, when the melting zone transforms into austenite completely, the cooling rate of austenite is proportional to the laser scanning speed and inversely proportional to laser power. It should be noted that the size limitation of sample will affect the cooling rate as well. When the scanning speed is low and the laser power is high, the heat transforming into substrate could not diffuse into the surrounding immediately, so the substrate temperature increases during laser processing, which decrease the temperature gradients and make the cooling rate of molten pool lower. Moreover, when the laser scanning speed is lower than 5 mm/s, the area size of molten zone increases sharply following the increase of laser power, which will lead to the lower cooling rate in the center part of molten pool. The reduction of cooling rate could weaken the effect of martensite reinforcement and cause the hardness of molten zone changed with different laser parameters, which is exhibited in Fig. 3.

As the results shown in Fig. 5, laser parameters influences the spectral intensity complexly and the change rule shows some difference with the previous work^12^. The spectral line intensity can be described as^28^:

4 $$I=\frac{hc{N}_{0}gA}{4\pi \lambda Z}\exp [-\frac{E}{kT}]$$

where *h* means Planck’s constant, *c* is speed of light, *λ* is wavelength, *g* is degeneracy, *A* is the transition probability (Einstein A coefficient), *N*_0_ is the total species population, *Z* is the partition function usually taken as the statistical weight of the ground state, k is Boltzmann constant, *T* is plasma temperature and *E* is upper energy level.

It follows from Eq. (4) that the intensity emitted at a frequency is dependent upon number density of a species at the ground state, the depth of plasma domain and the absolute temperature. For a special spectral line, the frequency of emission can be regarded as constant. The position of detecting sensor is fixed during the whole experiment, so the influence of depth of plasma domain can be ignored. Therefore, the emission intensity is related to concentration of the species at the ground state and the plasma temperature. For the plasma temperature, it is proportional to the laser power input^15^. For the species at the ground state, it is influenced by the vaporization rate of species. The vaporization flux for this element i from the molten pool surface at low pressure is given by the following equation^32^:

5 $${J}_{i}(T)=44.34\frac{{P}_{i}(T)}{\sqrt{{M}_{i}T}}$$

where *J*_*i*_(*T*) is flux of element i, *P*_*i*_(*T*) is partial pressure of element i over the alloy at T K (atm), *M*_*i*_ is molecular weight of element i and *T* is temperature.

For the vapor pressure of iron and chromium, they can be written as

6 $$log{P}_{Fe}=-\,24.609\times \frac{{10}^{3}}{T}-8.321logT+0.668\times {10}^{-3}T-0.305\times {10}^{-7}{T}^{2}+38.003$$

7 $$log{P}_{Cr}=-\,13.505\times \frac{{10}^{3}}{T}-33.658logT-9.290\times {10}^{-3}T-8.381\times {10}^{-7}{T}^{2}-87.077$$

If the surface renewal rates were assumed to be dominant and there was no element segregation on the surface of molten pool, representing the surface of the molten pool approximately by a circle of radius r, the following equation gives the vaporization rate *v*_*vapor*_^33^:

8 $${v}_{vapor}=\sum _{element}\,{\int }_{0}^{r}2\pi {J}_{i}(T)rdr$$

It can be inferred from Eq. (5) to Eq. (8), for both Cr and Fe, the vapor rate will rise with the increase of temperature of molten pool. The temperature of molten pool is proportional to the input power per unit time:

9 $$T\propto \frac{P}{v}$$

Therefore, the molten pool temperature will rise with the increase of laser power or the reduction of laser scanning speed, correspondingly, the variation of vapor rate of metal gas shows the same trend with the change of laser parameters.

When the molten pool temperature reach to the boiling temperature of alloy, the surface temperature of a molten pool would not increase with an increasing input energy, for a steady situation, the vaporization rate for a molten pool with boiling temperature can be written as^34^;

10 $${v}_{vapor}=\frac{P}{\rho ({L}_{v}+{C}_{p}({T}_{v}-{T}_{0}))}$$

where *P* is laser power density, *ρ* is metal density, *L*_*v*_ is latent heat of vaporization, *C*_*p*_ is heat capacity, *T*_*v*_ is vaporization temperature (boiling temperature) and *T*_0_ is ambient temperature. When the molten pool temperature attains to boiling point, *ρ*, *L*_*v*_, *C*_*p*_, *T*_*v*_, *T*_0_ can be set as constants and it means the vaporization rate is proportional to the input power per unit time as well.

Therefore, the relative mean intensity of spectral lines should increase with the enhance of laser power density per unit time. However, as the results shown in Fig. 5, when the laser scanning speed is lower than 5 mm/s, the variation of relative mean intensity do not follow the above relationship. The main reasons can be summarized as that the variation of spectral line intensity is not just influenced by input laser power density but also affected by the diffusion process of metal vapor from molten pool. As the Maxwell-Boltzmann distribution shown^35^:

11 $$f(v)=\sqrt{{(\frac{m}{2\pi kT})}^{3}}4\pi {v}^{2}{e}^{\frac{m{v}^{2}}{2kT}}$$

where *m* is the particle mass, *k* is the Boltzmann’s constant, *T* is thermodynamic temperature and *v* is particle speed. Depending on the Eq. (11), the most probable thermal velocity of the species corresponding to most probable kinetic energy distribution as per the Maxwell’s velocity distribution is:

12 $$v\propto {(\frac{kT}{m})}^{\frac{1}{2}}$$

It can be deduced from Eq. (12) that the most probable velocity of metal gas decreases with the particle’s temperature reduction. In a real laser processing, the high laser energy would accelerate the process of vaporization on the molten pool surface. Due to the mass removal from molten pool surface, the melt level under the laser beam becomes lower. In the case of the normal (Gaussian) radiation intensity distribution in the focal spot, the temperature distribution can be^15^:

13 $$T(x,y,z,t)={T}_{0}+\frac{\alpha P}{2\pi \kappa \sqrt{4\pi \chi }}\times {\int }_{0}^{t}\frac{dt}{\sqrt{t}(t+\frac{{r}_{f}^{2}}{4\chi })}exp[-\frac{{(x+vt)}^{2}+{y}^{2}}{4\chi (t+\frac{{r}_{f}^{2}}{4\chi })}-\frac{{z}^{2}}{4\chi t}]$$

where *P* is the total laser beam power; *v* is the laser scanning speed along x-axis; *α* is the absorption coefficient; *r*_*f*_ is the focal beam radius and *χ* is the thermal diffusivity.

When a laser beam with Gaussian energy profile is applied in a laser processing, the temperature at center of molten pool could be higher than that at the edge. If the input energy per unit time is high enough, there will form a concave surface in the center of molten pool, correspondingly, if the detecting location of plasma signal above the molten pool is in a fixed position, the diffusion distance of metal vapor from the concave surface to detecting point can be lengthened. As the diffusion distance becomes longer, more energy of the metal vapor transforms into the surrounding area, and the temperature of metal vapor reduces to a lower level. Therefore, the most probable velocity of metal gas declines for the temperature decrease of metal gas, and the particle flux passing the detecting point becomes weak per unit time, which cause the peak intensity of spectrum reduced. In conclusion, when the laser scanning speed is higher than 10 mm/s, the intensity of spectral lines increases with the increase of laser power, however, when the laser scanning speed is low, for the shape effect of molten pool surface, the relative intensity shows a decreasing trend after its original increase.

It should be noted that, in terms of atomic lines, the line intensity increases with the increase of plasma temperature firstly and then reduces due to the enhancement of the degree of ionization, whereas, the ionic lines intensity enhances with the increase of plasma temperature and population density of the ground state until secondary ionization happens^36^. Therefore, the degree of ionization about plasma could affect the variation about relative mean intensity of atomic spectral lines as well. By the comparison between NIST database and the spectra shown in Fig. 2, there is no ionized spectral lines in the spectrum, which means when the laser power density is around 10^5^–10^6^ W/cm^2^ (used in this experiment), the ionization degree of plasma induced by laser is weak and the effect of ionization degree on the intensity of spectral lines can be ignored in this experiment.

Based on the above analysis, it can be found the micro-hardness is decided by the phase in the molten zone. the transition of phase depends on the cooling rate of molten zone. according to the relation shown in Eq. 3, the cooling rate is strongly associated with three factors: laser power, laser scanning speed and thermal conductivity of substrate respectively. Correspondingly, the parameters of laser processing and the thermophysical properties can affect the intensity of spectral lines. The heating temperature can decide the metallic atom density in plasma, and the emission energy is from the laser power, in addition, the collection of spectrometers is related to the laser scanning speed. Hence, a bridge, combining with laser parameters and thermophysical parameters, need be established to correlate the spectra information with micro-hardness of molten zone.

To build up the bridge, the dimensionless group is introduced in this experiment^37^. The dimensionless power and dimensionless velocity can be expressed by: ${P}^{\ast }=\frac{4AP}{\pi {h}_{s}v{D}^{2}}$ and ${{\rm{v}}}^{\ast }=\frac{vD}{2\alpha }$ respectively^38,39^, therefore, the dimensionless energy intensity can be shown as:

14 $${\rm{\delta }}=\frac{dimensionless\,power}{dimensionless\,velocity}=\frac{\frac{4AP}{\pi {h}_{s}v{D}^{2}}}{\frac{vD}{2\alpha }}=\frac{8A\alpha P}{\pi {v}^{2}{h}_{s}{D}^{3}}=\frac{8A\alpha P}{\pi {v}^{2}\rho {C}_{p}{T}_{m}{D}^{3}}=8\frac{A\alpha }{\pi \rho {C}_{p}{T}_{m}}\cdot \frac{P}{{v}^{2}{D}^{3}}$$

where *P* is the laser power (W); *D* is the laser beam diameter (m); *v* is the laser scanning speed (m/s); *A* is the absorption rate, *ρ*, *α*, *C*_*p*_, *T*_*m*_ are the density (kg/m^3^), thermal diffusivity (m^2^/s), specific heat capacity (J/kg·K) and the melting point of the substrate (K). The ratio $\frac{A\alpha }{\rho {C}_{p}{T}_{m}}$ is the effect from thermophysical properties of substrate. $\frac{P}{{v}^{2}{D}^{3}}$ is the influence from laser manufacture parameter.

For the variables of temperature dependence, such as ρ, α, C_p_, they are changeable during heating process. Therefore, a simple corrected parameter $\beta =\frac{D{\prime} }{D}$ is introduced into dimensionless energy intensity, and the dimensionless energy intensity can be rewritten as:

15 $${\delta }^{\ast }=8\frac{A\alpha \beta }{\pi \rho {C}_{p}{T}_{m}}\cdot \frac{P}{{v}^{2}{D}^{3}}=8\frac{A\alpha }{\pi \rho {C}_{p}{T}_{m}}\cdot \frac{P}{{v}^{2}{D}^{2}D{\prime} }$$

where D’ is the real width of molten zone.

To the intensity of Fe I spectral lines, considering the effect of surface shape of molten zone, the dimensionless spectral lines intensity can be corrected as:

16 $${I}^{\ast }=\frac{{I}_{relative}}{ln(\frac{S}{S{\prime} })}$$

where *S*’ is the cross-section area size of molten zone and *S* is cross-section area size of whole sample (m^2^), I_relative_ is the relative intensity of spectral lines detected in the experiment.

**Figure 6:**
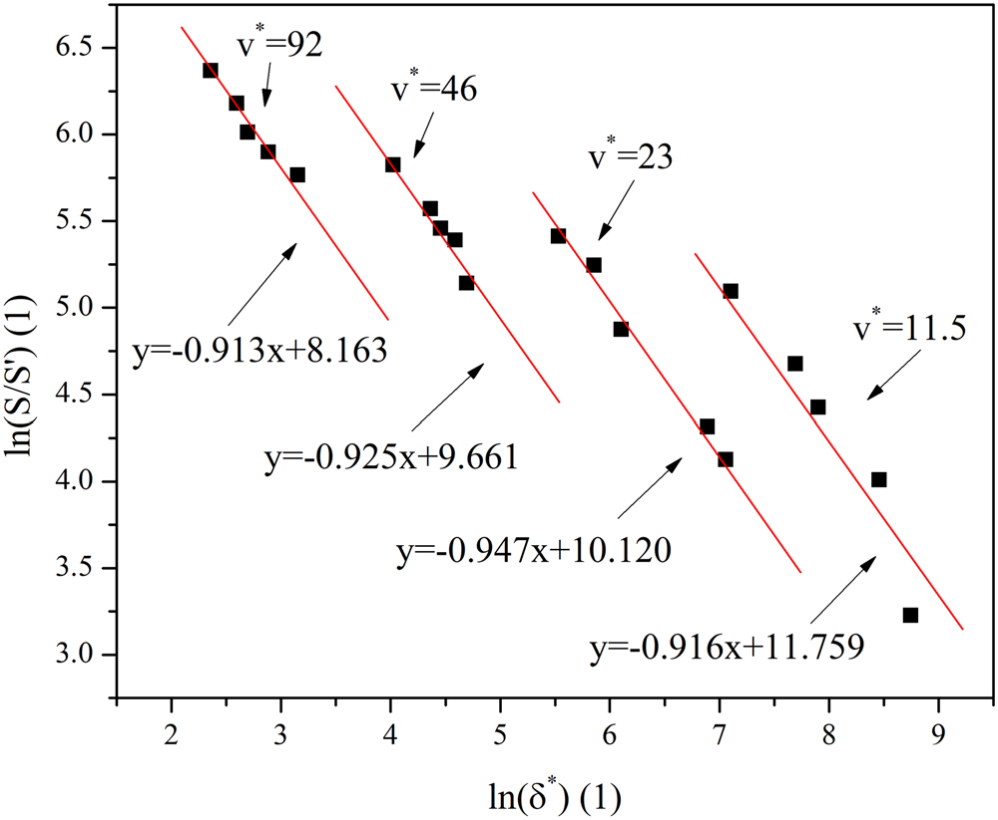
The correlation between dimensionless energy density and corrected parameter of dimensionless intensity.

Figure 6 shows the correlation between δ^*^ and $ln(\frac{S}{S{\prime} })$, according to the data shown in Fig. 6, it can be deduced that $ln(\frac{S}{S{\prime} })$ can be approximatively expressed as:

17 $$ln(\frac{S}{S{\prime} })\approx -0.926\,\mathrm{ln}({\delta }^{\ast })-0.051{v}^{\ast }+12.245$$

**Figure 7:**
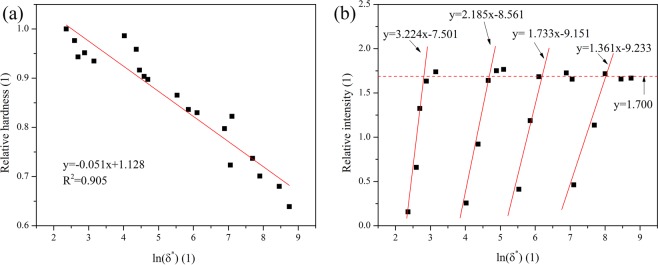
The correlation among dimensionless micro-hardness, dimensionless intensity of spectra lines and dimensionless energy density (**a**) dimensionless micro-hardness of molten zone and dimensionless energy density (**b**) dimensionless intensity of spectral lines and dimensionless energy density.

To satisfy with the requirement of dimensionless analysis, the dimensionless micro-hardness is used in this experiment, it is calculated by the ratio of present micro-hardness to a standard value. The standard value is the micro-hardness of molten zone prepared with the smallest laser power and the fastest laser scanning speed in this experiment. The relationship between dimensionless micro-hardness and dimensionless energy density is shown in Fig. 7(a) and the correlation between dimensionless intensity of Fe I spectral lines and the dimensionless energy density is shown in Fig. 7(b).

It can be found there is an approximate linear correlation between dimensionless hardness and ln(δ^*^), the coefficient is 0.905. When the dimensionless velocity is fixed, at first, there is a linear correlation between ln(δ^*^) and dimensionless intensity of Fe I spectral lines, then the relative intensity becomes stable with the continuous increase of ln(δ^*^). Therefore, the correlation of relative intensity and dimensionless energy density can be expressed by:

18 $${I}^{\ast }=\{\begin{array}{ll}{f}_{1}({v}^{\ast })\mathrm{ln}({\delta }^{\ast })+{f}_{2}({v}^{\ast })\, & ln({\delta }^{\ast })\le \frac{C-{f}_{2}({v}^{\ast })}{{f}_{1}({v}^{\ast })}\\ C & ln({\delta }^{\ast })\ge \frac{C-{f}_{2}({v}^{\ast })}{{f}_{1}({v}^{\ast })}\end{array}$$

where *f*_1_(v^*^) and *f*^2^(v^*^) are the functions used dimensionless velocity as independent variable.

Depending on the data and correlation shown in Fig. 7(b), *f*_1_(v^*^) and *f*_2_(v^*^) can be simplified as linear function, and dimensionless intensity of an element spectral lines I* can be written as:

19 $${I}^{\ast }=\{\begin{array}{ll}(a{v}^{\ast }+b)ln({\delta }^{\ast })+(c{v}^{\ast }+d) & ln({\delta }^{\ast })\le \frac{C-(c{v}^{\ast }+d)}{a{v}^{\ast }+b}\\ C & ln({\delta }^{\ast })\ge \frac{C-(c{v}^{\ast }+d)}{a{v}^{\ast }+b}\end{array}$$

According to the fitting results shown in Fig. 7(a), the correlation between dimensionless hardness H^*^ and dimensionless laser density *ln*(δ^*^) can be expressed as:

20 $$ln({\delta }^{\ast })=-19.608\,{H}^{\ast }+22.118$$

By non-linear fitting function offered with MATLAB 2016b, the calculation results are: a = 0.023, b = 1.151, c = 0.022, d = −9.575 and C = 1.700 and the coefficient is 0.891. By plugging Eqs. (16), (17) and (20) into Eq. (19), The final correlation between spectra intensity and micro-hardness of molten zone can be summarized as:

21 $$\begin{array}{c}\frac{{I}_{relative}}{-0.926\,\mathrm{ln}({\delta }^{\ast })-0.051{v}^{\ast }+12.245}\\ =\{\begin{array}{cc}(\,-\,0.451{v}^{\ast }-22.596){H}^{\ast }+(0.531{v}^{\ast }+15.883) & ln({\delta }^{\ast })\le \frac{1.700-(0.022{v}^{\ast }-9.575)\,}{0.023{v}^{\ast }+1.151}\\ 1.700 & ln({\delta }^{\ast })\ge \frac{1.700-(0.022{v}^{\ast }-9.575)\,}{0.023{v}^{\ast }+1.151}\end{array}\end{array}$$

It can be found from Eq. (21) that there is a quantitative relation among the dimensionless intensity of spectral lines, dimensionless velocity and dimensionless micro-hardness of molten zone, which means that the micro-hardness of molten zone can be deduced with the intensity of laser induced spectra and the laser scanning speed.

**Figure 8:**
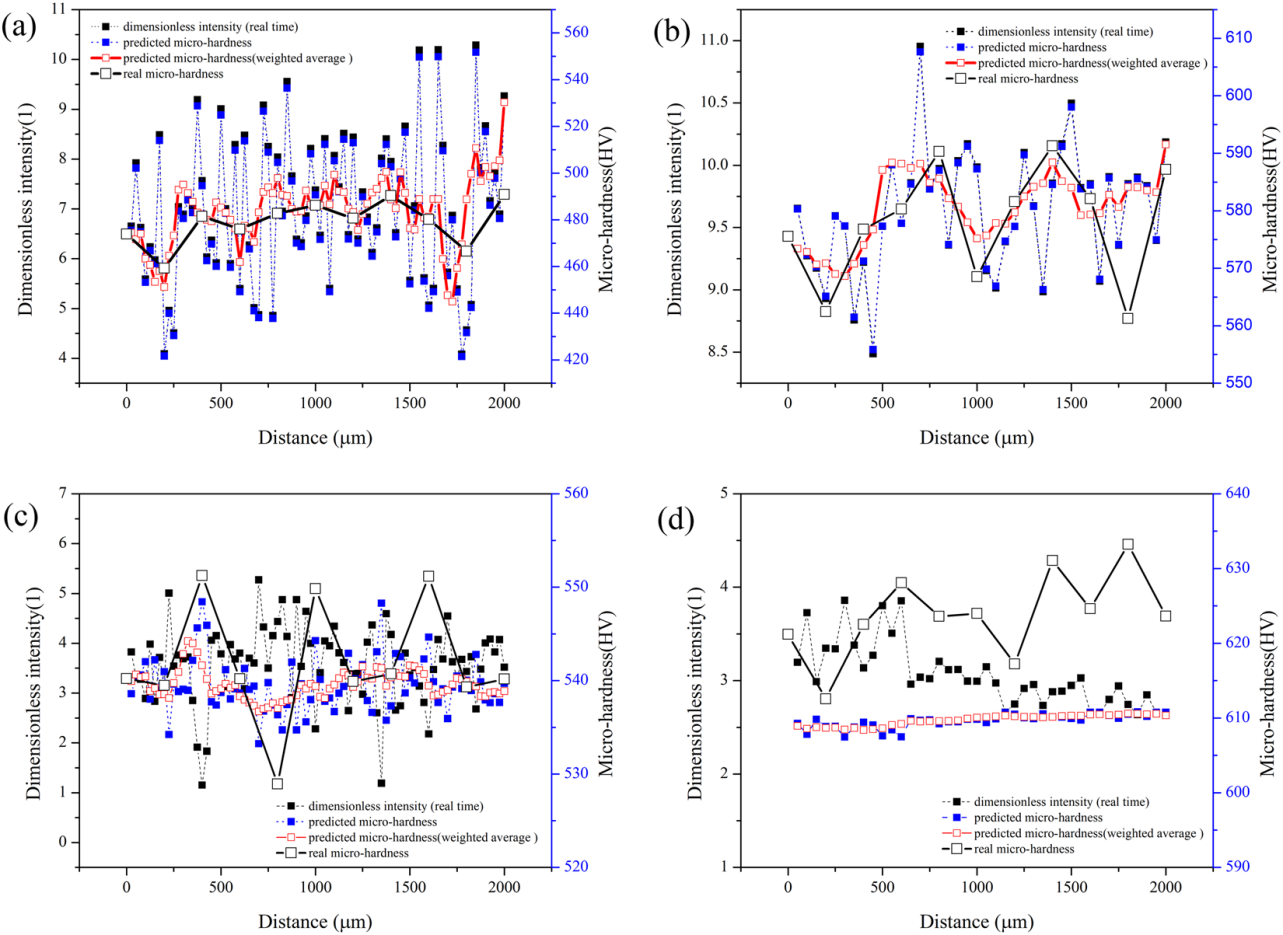
On-line monitoring results and real hardness of molten zone under different laser parameters (**a**) 1700 W and 5 mm/s; (**b**) 1700 W and 10 mm/s; (**c**) 1100 W and 5 mm/s; (**d**) 1100 W and 10 mm/s.

To test the error of monitoring system, the new laser parameters are used in experiment. Figure 8 shows the prediction of micro-hardness of molten zone, which is deduced with Eq. (21). The signal data is collected in the middle of the samples. Considering the fluctuation of spectral signal, the original prediction micro-hardness is smoothed by the weighted average of each 10 adjoining points, and the smoothed data is marked with red empty points. Moreover, the real micro-hardness of molten zone surface is tested and shown with black empty points. By comparison between the prediction results and real micro-hardness of samples, it can be calculated that the mean errors of this monitoring system are 1.60% (1700 W, 5 mm/s), 3.09% (1700 W, 10 mm/s), 2.31% (1100 W, 5 mm/s) and 2.25% (1100 W, 10 mm/s) respectively.

## Conclusion

In this work, the emission spectra were detected during a real laser melting process, the change rules about the micro-hardness of molten zone and the intensity of emission spectra were researched. The main conclusion can be summarized as:

The micro-hardness of molten zone shows a decrease trend with the increase of laser power, or an increase trend with reduction of laser scanning speed; when the laser scanning speed is lower than 5 mm/s or the laser power is higher than 1400 W, the hardness of molten zone shows a significant variation with the change of laser processing parameters, on the contrary, the variation of hardness is slight. The main reason of micro-hardness variation is attributed to cooling rate. With the decrease of cooling rate in molten pool, the microstructure change rule is shown as follows: Martensite and lower Bainite → lower Bainite and upper Bainite → Ferrite and granular Bainite. In a dimensionless system, where the dimensionless number is deduced with laser parameters and thermos-physical parameters, there is a linear correlation between dimensionless micro-hardness of molten zone and dimensionless energy density.

The correlation between spectral intensity and laser parameters are non-monotonic. When the laser scanning speed is lower than 5 mm/s, for both Fe I and Cr I spectral lines, with the enhance of laser power, the relative intensity shows a declining trend after its original increase. The reasons for variation of spectra are combined effects among plasma temperature, number density of a species at the ground state and shape of molten pool surface. When the scanning speed remains fixed, with the increase of dimensionless energy density, the dimensionless intensity of spectral lines becomes stable after its original increase.

By the method of non-linear regression, the correlation among dimensionless intensity of Fe I spectral lines I_relative_, dimensionless micro-hardness and dimensionless velocity was established, and a monitoring system of micro-hardness of molten zone was built by the quantitative relation between dimensionless intensity of Fe I spectral line and dimensionless of micro-hardness. For checking the accuracy of the monitoring system, 4 new groups of laser parameter were used to remelt AISI 4140 steel surface. The monitoring results indicate, comparing the predicting hardness and real hardness, the max relative error was 3.09%, and the minimum relative error was 1.60%.

It means that the emission spectrum can be an approach to monitor the properties associated with micro-hardness of a part which is manufactured by laser process.

## Data Availability

The data used to support the findings of this study are available from the corresponding author upon request.
